# Bioelectrical impedance and lung function—associations with gender and central obesity: results of the EpiHealth study

**DOI:** 10.1186/s12890-024-03128-0

**Published:** 2024-07-04

**Authors:** Mikaela Qvarfordt, Erik Lampa, Gui-Hong Cai, Lars Lind, Sölve Elmståhl, Magnus Svartengren

**Affiliations:** 1 Department of Medical Sciences, Occupational and Environmental Medicine, Uppsala University, Uppsala, Sweden; 2 Department of Laboratory Medicine, Clinical Physiology, Karolinska Institute, Stockholm, Sweden; 3 Department of Medical Sciences, Cardiovascular Epidemiology, Uppsala University, Uppsala, Sweden; 4 Division of Geriatric Medicine, Department of Clinical Sciences in Malmö, Lund University, Malmö, Sweden

**Keywords:** Spirometry, Body composition, Bioelectrical analysis, Lung function diagnostics, Fat mass

## Abstract

**Background:**

Obesity is a major public health concern associated with various health problems, including respiratory impairment. Bioelectrical impedance (BIA) is used in health screening to assess body fat. However, there is no consensus in healthcare on how body fat should be assessed in relation to lung function. In this study, we aimed to investigate how BIA in relation to waist circumference contribute, using data from a large Swedish population study.

**Methods:**

A total of 17,097 participants (aged 45–75 years) were included in the study. The relationships between fat mass, waist circumference, and lung function were analysed using weighted quantile sum regression.

**Results:**

Increased fat mass was significantly associated with decreased lung function (FEV1, FVC) in both sexes. Also, the influence of trunk fat and waist circumference on FVC and FEV1 differed by sex: in males, waist circumference and trunk fat had nearly equal importance for FVC (variable weights of 0.42 and 0.41), whereas in females, trunk fat was significantly more important (variable weights 0.84 and 0.14). For FEV1, waist circumference was more important in males, while trunk fat was more significant in females (variable weights male 0.68 and 0.28 and 0.23 and 0.77 in female).

**Conclusions:**

Our results suggest that trunk fat should be considered when assessing the impact of adipose tissue on lung function and should potentially be included in the health controls.

**Supplementary Information:**

The online version contains supplementary material available at 10.1186/s12890-024-03128-0.

## Introduction

Obesity is associated with various health problems and is a major public health concern [1–3]. Previous studies have clearly established an association between obesity and several lung impairments, such as a higher risk of reduced lung volume, asthma, sleep apnoea, or chronic obstructive pulmonary disease (COPD) [4–9]. Excessive adipose tissue affects the respiratory system through various mechanisms, for example by increasing intrathoracic pressure due to fat accumulation in the abdomen, which influences lung expansion and intrathoracic pressure. This leads to a reduction in tidal volume, increased respiratory rate and an overall reduction in lung volume [10, 11]. Adipose tissue can also affect the respiratory system on a cellular level, e.g. by increasing the general level of inflammation in the body, but the mechanisms are not fully understood [7, 11–13]. In addition, there are a gender-specific differences in the distribution of adipose tissue in the body that should be considered. Fat distribution patterns can either be described as a central body fat pattern, i.e. abdominal obesity, which is the accumulation of fat in the abdomen, thorax, and visceral organs. In the peripheral body fat pattern, fat accumulates in the upper extremities, i.e. in the hips, thighs, legs, and arms, as well as the subcutaneous tissue. Central obesity is more common among male and peripheral obesity more common among women [14, 15]. It is therefore reasonable to assume that lung function is affected differently by adipose tissue in male and female, although this has not always been clear in previous studies [5, 9, 11].

In healthcare, obesity and body composition are measured as part of health status assessment. Currently, there is no generally recognised method for measuring obesity to predict lung function impairment. Although body mass index (BMI) is possibly one of the most commonly used methods for evaluating body composition, the method has clear limitations in assessing body fat distribution and cannot distinguish between fat mass and lean mass [16, 17]. Studies have shown that both elevated and low BMI levels have been associated with reduced lung function in previous studies [6, 18]. For example, the lungs form during childhood, and it is evident that larger body size, as indicated by BMI, is associated with better lung function [19]. This effect is likely to persist initially; however, once the lungs are fully developed, the dynamic changes and an increase in BMI is then more closely associated with weight gain, predominantly as adipose tissue [18]. Previous studies have shown that increased waist circumference is more strongly associated with reduced lung function than BMI [10]. Measurement of waist circumference is recommended in the assessment of obesity and is included in the definition of metabolic syndrome, given that it is performed accurately [20]. Although there are guidelines describing how to correctly measure waist circumference correctly, it is unclear how well this procedure is implemented in healthcare settings and studies have shown a lack of accuracy in measurement [21, 22]. Also, previous studies have shown that increased body fat percentage (BF%) has a negative impact on lung function, even in individuals with normal weight to slightly overweight [23, 24]. There are therefore reasons to investigate whether there is an alternative method of measuring body fat and body composition in relation to lung function. For example, more advanced methods of assessing body composition, such as bioelectrical impedance analysis (BIA) or dual-energy X-ray absorptiometry (DXA), can differentiate between fat and other tissues [25, 26]. BIA assesses body composition by estimating and differentiate fat distribution in different areas of the body, including the arm-, leg- and trunkfat [26, 27]. Trunk fat measures all the fat in the trunk region, making it a stronger indicator of central obesity. DXA is considered the standard for assessing body composition [16]. However, the BIA method has several practical advantages, it does not require radiation, is an relatively inexpensive and more accessible method, does not require training, and is easy to standardise [27–29]. The BIA method is used in health assessment to evaluate obesity, although it is rarely associated with impaired lung function. There are few studies linking the BIA method to impaired lung function. Nevertheless, they have shown a negative association with lung function [30, 31].

Given the advantages of the BIA method, the aim of our study was to investigate the relationship between BIA and lung function in relation to waist circumference in middle-aged and elderly subjects. For this purpose, we used data from the Swedish cohort study EpiHealth (www.epihealth.se) [32], a large population study.

## Material and methods

### Participants

**Fig. 1:**
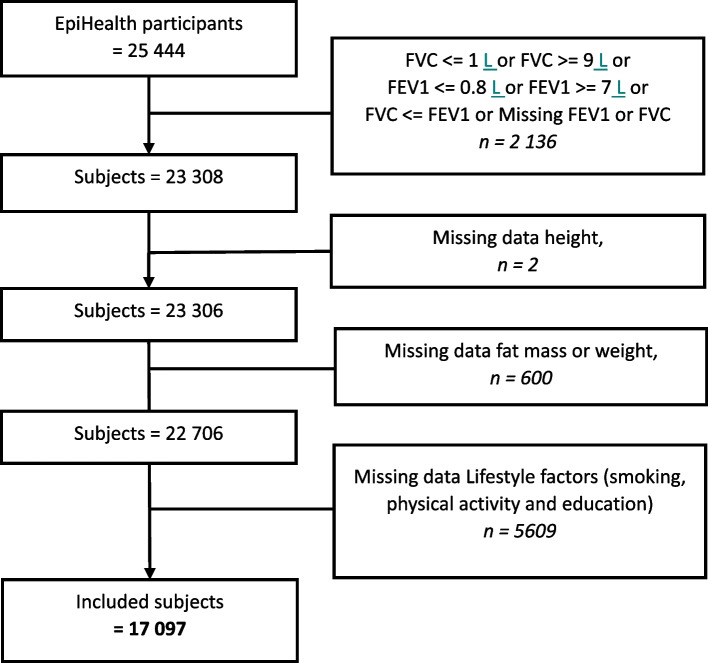
Flow chart of missing data and included subjects. Limits for FEV1 and FVC are presented in Liters (L)

In this study, we used data from the EpiHealth study. The study started in 2011 and included a questionnaire and a visit to a test centre located in Malmö or Uppsala, with the aim of studying the connections between lifestyle factors and genetic predisposition contributing to the development of the most common diseases, such as cardiovascular and respiratory [32]. The initial sample size was 25,444 participants (aged 45–75 years). In this study, subjects with missing data on weight, height, lifestyles factors, bioimpedance, or extremely improbable lung function data (with forced expiratory volume in 1 s (FEV1) < 0.8 L or > 7 L and forced vital capacity (FVC) < 1 L or > 9 L) were excluded from the study, resulting in 17 097 remaining for the analysis (see Fig. 1). The study was approved by the Ethics Committee of Uppsala University (Dnr 2010/402), and all participants provided written informed consent for participation. The study was performed in accordance with the ethical standards of the responsible committee and with the most recent amendment of the Declaration of Helsinki, 1975.

### Bioimpedance, body mass index, and waist circumference

In the Epihealth study, the subjects underwent a series of body measurements, including height (cm), waist- and hip circumference (cm), weight (Kg), BIA (arm, leg and trunk fat in Kg), and spirometry (FVC, FEV1 in Liters and z-score). The Tanita BC-418MA segmental body composition analyser was used to measure fat mass. The subject stood with electrodes placed in each hand and under the feet to calculate the total body mass, divided into segments, extremities, and torso [32].

Waist circumference was measured with a measuring tape in a standing position and at the end of normal expiration, midway between the lower costal margin and the anterior superior iliac crest.

### Lung function

Lung function tests were performed following internationally accepted guidelines using a MiniSpir spirometer (Medical International Research, Waukesha, WI, USA). The subject made at least three and at most nine attempts. Forced vital capacity (FVC) and forced expiratory volume in 1 s (FEV1) were measured; moreover, predicted values and z-scores were calculated using the 2012 Global Lung Function Initiative (GLI) equations [33].

### Lifestyle factors

The lifestyle factors pertaining to smoking habits, physical activity, and educational status from the questionnaire were included in the analysis. The level of education was grouped according to elementary school, upper secondary school, or university. Smoking habits were defined as: non-smoker, ex-smoker, or current smoker at the time of the survey. The subjects' physical activity during leisure time was categorised into three groups based on their answers on a seven-point scale: low level (sedentary activity), medium level (physical activity of at least one to two hours per week) or high level of physical activity (strenuous activity of at least three hours per week). A low level of physical activity, defined as a score of one, included predominantly sedentary activities, occasional walking, or light housework. The moderate level, scored as two, included activities such as regular walking or light physical activity for up to 2 h per week. A high level of physical activity, scored three, indicated vigorous exercise for at least 3 h per week. This categorisation scheme was adopted from Byberg et al. [34]. The EpiHealth study and its questionnaire are described in Lind et al. 2013 [32].

### Statistical analyses

The relationships between fat mass, waist circumference, and lung function were analysed with Weighted Quantile Sum Regression (WQS) [35], which is a method suitable when dealing with highly correlated variables. (see S1a, b). The model constructs an index equal to a weighted sum of variables that are highly correlated with each other. The index is tested as a whole against the outcome (FEV1, or FVC).

WQS estimates the association between the outcome and a index of percentiles with variable weights determined empirically from the data, possibly adjusting for other covariates, using a generalised linear model of the form:

$g\left(\mu \right)={\beta }_{0}+{\beta }_{1} {\sum }_{i = 1}^{c}{w}_{i}{q}_{i} + {z}{\prime}\varphi$ subject to ${\sum }_{i = 1}^{c}{w}_{i} = 1$ and $0\le {w}_{i}\le 1$

Where $g\left(.\right)$ is the link function, i.e. the identity link when the outcome variable is continuous, and ${w}_{i}$ is the variable weight parameter associated with the *i*^th^ component with percentile values ${q}_{i}$. The model is thus a linear model with the data derived index as one of the independent variables. All exposure variables are grouped into decile groups, so ${\beta }_{1}$ corresponds to an increase in the entire index of 10%.

The index variable, henceforth called the BIA/waist index, was formed using all the BIA variables in addition to the waist circumference. Each BIA variable and waist circumference was divided info ten groups according to the sex specific deciles. 40% of the data was used to train the model and optimize the variable weights. Once the variable weights were estimated, the BIA/waist index was formed, and the regression model was fitted in the 60% of the data set aside for testing. For example, if the variable weights for the BIA/waist index was found to be 0.75, 0.20, 0.02, 0.01, 0 and 0 for trunk fat, waist circumference, left and right arm and left and right leg, and the decile group values for those variables for an individual are 5, 6, 3, 3, 2, 2—the value of the BIA/waist index is calculated as 0.75 * 5 + 0.20 * 6 + 0.02 * 3 + 0.01 * 3 + 0 * 2 + 0 * 2 = 5.04.

The outcomes measured were FVC and FEV_1,_ and other variables in the model were weight in kg, height^−2^ in cm^−2^, the interaction between weight and height^−2^ which is equal to BMI [36], age in years, education, physical activity during free time, and smoking status (never, former, current).

To compare the coefficients between mean and women despite the different distributions of the BIA/waist index between the sexes, the coefficients were scaled by the sex specific interquartile range (IQR) of the BIA/waist index. This makes the coefficients reflect the same relative increase and are thus comparable.

All analyses were performed using R, a free software for statistical computing and graphics with the gQWS add-on package [37].

## Results

### Participant data

Participants' demographic data of the 17 097 included participants (94% were of European descent) were organised based on BF% and divided into quartiles, presented in Table 1. The mean age of the participants was 60.3 (SD ± 8.5) years, and 43% were male. According to the BMI, nearly the same proportions of men and women were obese (BMI⩾30), namely 17% of the men and 15% of the women. However, women had a greater body fat mass (measured using BIA) than men and more fat mass on the legs (and hip); see Table 2.

**Table 1:** Characteristics of the study participants in total and categorised according to quartile of body fat per cent (BF%)

Characteristic, grouped into quartiles by BF%		Total *N* = 22,706	Quartile 1 range (3.6 – 25.1) *N* = 5698	Quartile 2 range (25.1 – 30.8) *N* = 5758	Quartile 3 range (30.8 – 36.9) *N* = 5586	Quartile 4 range (36.9 – 58.6) *N* = 5664
**Gender**	Female	12,788 (56%)	785 (14%)	2251 (39%)	4251 (76%)	5501 (97%)
	Male	9918 (44%)	4913 (86%)	3507 (61%)	1335 (24%)	163 (2.9%)
**Age category**	45–55	6764 (30%)	2057 (36%)	1711 (30%)	1608 (29%)	1388 (25%)
	55–64	7395 (33%)	1832 (32%)	1850 (32%)	1833 (33%)	1880 (33%)
	65–75	8547 (38%)	1809 (32%)	2197 (38%)	2145 (38%)	2396 (42%)
**Smoking**	Never	6091 (35%)	1668 (41%)	1514 (34%)	1434 (33%)	1475 (33%)
	Former	9493 (55%)	1970 (49%)	2473 (56%)	2466 (57%)	2584 (57%)
	Current	1743 (10%)	411 (10%)	418 (9.5%)	440 (10%)	474 (10%)
	Missing	5379	1649	1353	1246	1131
**Physical activity**	Low	1309 (5.9%)	259 (4.7%)	371 (6.6%)	306 (5.6%)	373 (6.8%)
	Medium	16,638 (76%)	3546 (64%)	4060 (73%)	4345 (80%)	4687 (86%)
	High	4060 (18%)	1708 (31%)	1149 (21%)	783 (14%)	420 (7.7%)
	Missing	699	185	178	152	184
**Education level**	Elementary school	5973 (27%)	1174 (21%)	1498 (27%)	1519 (28%)	1782 (32%)
	High school	5898 (27%)	1568 (28%)	1465 (26%)	1350 (25%)	1515 (27%)
	Academic	10,334 (47%)	2809 (51%)	2658 (47%)	2614 (48%)	2253 (41%)
	Missing	501	147	137	103	114
**BMI category**	Underweight	566 (2.5%)	369 (6.5%)	163 (2.8%)	34 (0.6%)	0 (0%)
	Normal weight	8544 (38%)	2986 (52%)	2344 (41%)	2662 (48%)	552 (9.7%)
	Overweight	9843 (43%)	2258 (40%)	2582 (45%)	1988 (36%)	3015 (53%)
	Obese	3753 (17%)	85 (1.5%)	669 (12%)	902 (16%)	2097 (37%)

**Table 2:** Fat mass divided into trunk and extremities, shown in kilograms (kg). Female, *N* = 12,788 and Male, *N* = 9,918

Fat mass (kg)	Female, median (IQR)	Male, median (IQR)
Total body	24 (20 – 31)	21 (17 – 26)
Left arm	1.20 (0.90 – 1.60)	1.00 (0.80 – 1.30)
Right arm	1.10 (0.80 – 1.50)	1.00 (0.80 – 1.20)
Trunk	12.6 (9.8 – 16.0)	13.5 (10.6 – 16.6)
Left leg	4.70 (4.00 – 5.70)	2.80 (2.30 – 3.50)
Right leg	4.80 (4.00 – 5.80)	2.90 (2.30 – 3.60)
Waist circumference, cm	87 (79—95)	97 (91—104)
BMI, kg/m2	25.1 (22.8—28.2)	26.6 (24.5—29.0)
Body fat, %	36 (31—40)	25 (21—29)

### Lung function in relation to body fat percentage

Pulmonary function variables are presented as absolute values, z-score, and percentage of predicted values divided into quartiles based on BF%; see Table 5. Absolute and predicted values of both FEV1 and FVC as well as z-scores decreased across quartile groups of increasing BF%, while the ratio FEV1/FVC remained almost unchanged; see Table 3 and 4.

**Table 3:** Characteristics of the study participants in total and categorised according to quartile grouped into quartiles by body fat percentage (BF%). FEV1: forced expiratory volume in 1 s; FVC: forced vital capacity

Lung function grouped into quartiles by BF%	Total *N* = 22,706	Quartile 1 range [3.6 – 25.1) *N* = 5,698	Quartile 2 range [25.1 – 30.8) *N* = 5,758	Quartile 3 range [30.8 – 36.9) *N* = 5,586	Quartile 4 range [36.9 – 58.6] *N* = 5,664
FEV1 (L)	2.97 (2.48, 3.58)	3.66 (3.13, 4.18)	3.20 (2.73, 3.74)	2.76 (2.39, 3.17)	2.50 (2.17, 2.87)
FEV1 z-score	0.06 (-0.62, 0.77)	0.18 (-0.44, 0.91)	0.12 (-0.58, 0.83)	0.04 (-0.65, 0.73)	-0.09 (-0.79, 0.58)
FVC (L)	3.79 (3.15, 4.63)	4.75 (4.09, 5.47)	4.11 (3.50, 4.81)	3.51 (3.04, 4.07)	3.15 (2.72, 3.60)
FVC z-score	0.01 (-0.63, 0.71)	0.23 (-0.39, 0.96)	0.04 (-0.60, 0.77)	-0.02 (-0.66, 0.64)	-0.23 (-0.87, 0.45)
FEV1/FVC	0.79 (0.74, 0.83)	0.77 (0.73, 0.81)	0.79 (0.74, 0.82)	0.79 (0.75, 0.83)	0.80 (0.76, 0.84)
FEV1/FVC z-score	0.06 (-0.57, 0.63)	-0.10 (-0.73, 0.51)	0.07 (-0.57, 0.63)	0.07 (-0.55, 0.63)	0.19 (-0.42, 0.73)

**Table 4:** Characteristics of the study participants divided by gender and categorised into quartiles by body fat percentage (BF%). FEV1: forced expiratory volume in 1 s; FVC: forced vital capacity

**Lung function grouped into quartiles by BF% in male**	Total *N* = 9 918	Quartile 1 range [2.3—16.9) *N* = 2,512	Quartile 2 range [16.9—21.3) *N* = 2,500	Quartile 3 range [21.3—26.3) *N* = 2,452	Quartile 4 range [26.3—75.1], = 2,454
FEV1 (L)	3.62 (3.15–4.10)	3.75 (3.29–4.21)	3.63 (3.19–4.11)	3.58 (3.12–4.08)	3.49 (3.00–3.97)
FEV1z	0.07 (-0.60–0.80)	0.21 (-0.39–0.96)	0.16 (-0.54–0.86)	0.08 (-0.63- 0.81)	-0.17 (-0.87- 0.56)
FEV1.pred	101 (91–112)	103 (94–114)	102 (92–113)	101 (91–112)	97 (87–108)
FVC (L)	4.67 (4.09- 5.35)	4.91 (4.33- 5.55)	4.69 (4.15–5.34)	4.61 (4.03- 5.31)	4.46 (3.89- 5.11)
FVCz	0.05 (-0.62–0.77)	0.32 (-0.32–1.07)	0.12 (-0.51–0.82)	-0.02 (-0.65–0.71)	-0.29 (-0.95- 0.44)
FVC.pred	101 (91–111)	105 (96–116)	102 (92–112)	100 (90–110)	96 (86–106)
FEV1FVC	0.78 (0.73–0.82)	0.76 (0.72–0.81)	0.77 (0.73–0.82)	0.78 (0.73–0.82)	0.78 (0.74–0.83)
FEV1FVCz	0.04 (-0.61–0.66)	-0.18 (-0.77–0.44)	0.05 (-0.62- 0.65)	0.15 (-0.52- 0.72)	0.18 (-0.47- 0.80)
FEV1FVC.pred	100 (94–106)	98 (93–104)	100 (94–106)	101 (95–106)	102 (96–107)
**Lung function grouped into quartiles by BF% in female**	Total *N* = 12 788	Quartile 1 range [ 3.6–19.6) *N* = 3–210	Quartile 1 range [19.6–24.6) *N* = 3–227	Quartile 1 range [24.6–30.7) *N* = 3–183	Quartile 1 range [30.7–91.6] *N* = 3–168
FEV1 (L)	2.60 (2.26–2.96)	2.66 (2.31–3.03)	2.63 (2.28–2.97)	2.58 (2.25- 2.94)	2.54 (2.21–2.90)
FEV1z	0.05 (-0.63–0.75)	0.17 (-0.49–0.89)	0.10 (-0.54–0.77)	0.06 (-0.62- 0.76)	-0.11 (-0.81–0.55)
FEV1.pred	101 (91–111)	102 (93–113)	101 (92–111)	101 (91–111)	98 (88–108)
FVC (L)	3.28 (2.85–3.75)	3.37 (2.94–3.85)	3.32 (2.90–3.78)	3.27 (2.83–3.73)	3.17 (2.75–3.62)
FVCz	-0.02 (0.64–0.66)	0.17 (-0.44–0.89)	0.06 (-0.53–0.70)	-0.03 (-0.67–0.66)	-0.28 (-0.92–0.38)
FVC.pred	100 (90–110)	103 (93–113)	101 (92–111)	100 (90–110)	96 (86–106)
FEV1FVC	0.80 (0.75–0.83)	0.79 (0.75–0.83)	0.79 (0.75–0.83)	0.80 (0.75–0.83)	0.81 (0.77–0.84)
FEV1FVC.pred	101 (95–105)	99 (94–104)	100 (95–104)	101 (96–105)	102 (97–106)

### Associations between BIA, waist circumference, and lung function

Correlations between the different BIA variables and waist circumference were strong, with Spearman correlations ranging from 0.91 to 0.98 in men and 0.93 to 0.99 in women (supplemental Figures S1a and S1b illustrate the correlations). The BIA/waist index was related to decreased lung function in both men and women, as shown in Table 5, adjusted for height^−2^, weight, BMI, age, education, physical activity, and smoking.

**Table 5:** Estimated coefficients for an interquartile range increase in the BIA index with 95% confidence intervals

Outcome	Gender	Coefficient	95% CI
FVC	Male	-0.39	-0.48; -0.30
	Female	-0.25	-0.30; -0.19
FEV1	Male	-0.19	-0.26; -0.13
	Female	-0.18	-0.22; -0.13

**Fig. 2:**
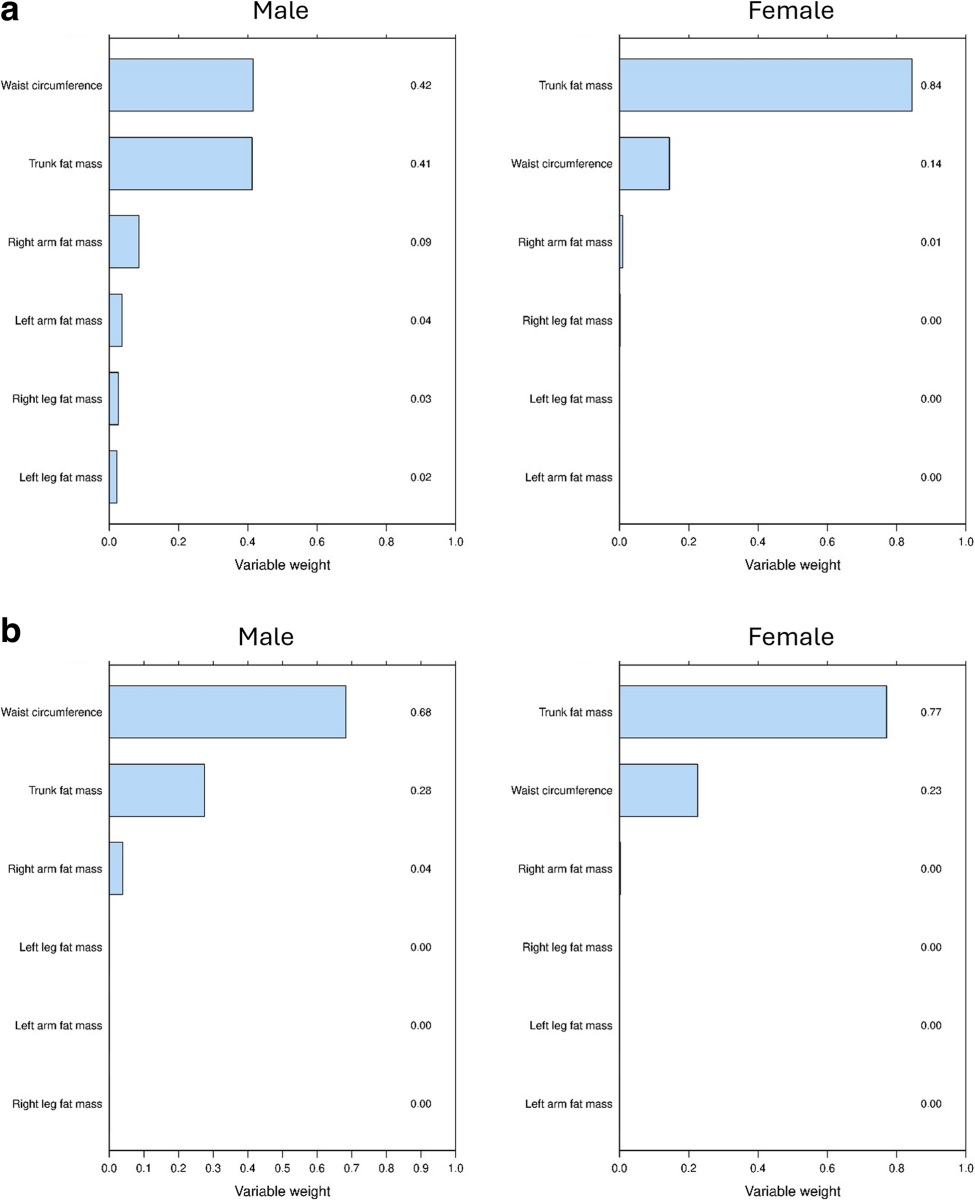
**a** Bar chart showing the estimated variable weights in the BIA/waist index using weighted quantile sum regression for Forced Vital Capacity (FVC) adjusting for height, weight, age, education, physical activity, and smoking. **b** Bar chart showing the estimated variable weights in the BIA/waist index using weighted quantile sum regression for Forced expiratory volume in 1 s (FEV1) adjusting for height, weight, age, education, physical activity, and smoking

The results of the statistics analysis using WQS estimated variable weights are shown in Figs. 2a and 2b, and boxplots of their bootstrap distributions are shown in supplemental Figure S2. The influence of trunk fat and waist circumference on FVC was different between men and women. For male waist circumference was as important as trunk fat in with estimated variable weights of 0.42 and 0.41 in women, trunk fat was clearly more important than waist circumference, with variable weights estimated as 0.84 and 0.14, respectively. Regarding FEV1, waist circumference was more important than trunk fat mass, variable weights 0.68 and 0.28, respectively, in male, with the situation reversed in women, with estimated variable weights 0.23 and 0.77 for waist circumference and trunk fat, respectively.

## Discussion

The purpose of this study was to investigate how BIA study the relationship between adipose tissue and lung function in relation to waist circumference, using data from a large population study—EpiHealth.

Our results showed that the BIA method is negatively associated lung function in both men and women. However, waist circumference provides information that is not captured by the BIA variables, particularly in men. This is most evident regarding reduced lung volume (measured as FVC), which is congruent with the results of previous research [5, 10]. This is most likely a consequence of the sex difference in fat distribution on the torso, that is, abdominal or gynoid obesity. Previous studies have shown that abdominal fat distribution has a strong association with reduced lung volume, which is consistent with our results [5, 6, 10]. Svartengren et al. (2020) showed that BMI is only related to reduced lung function in the presence of increased waist circumference, that is, increased waist circumference [10], which further proves that body constitution, hence, where fat is distributed, is essential. Abdominal obesity has shown to have a greater impact on metabolic inflammation since visceral fat is more metabolically active than subcutaneous fat. It also has a greater mechanical impact due to the limitation of chest expansion and descent of the diaphragm, and therefore, increased thoracic pressure during the FVC manoeuvre [6, 38]. In this study, we could not determine how much of the effect on lung function was due to mechanical impact or through substances secreted from the adipose tissue.

Regarding women, our results showed that it is trunk fat mass and not waist circumference that drives the negative association between adipose tissue and lung function, both regarding FEV1 and FVC. Thus, trunk fat rather than waist circumference could be a more important predictor of reduced lung function in women than waist circumference. This is especially interesting as waist circumference is a widely used measurement related to obesity-related health problems and also included in the definition of metabolic syndrome.

A few previous studies have investigated the association between total body fat per cent and lung function. As an example, Chen et al. showed that BF% measured by BIA showed an inverse relationship with lung function in normal weight subjects [23]. Wannamethee et al. showed not only that total body fat and increased waist circumference are inversely associated with lung function, but also that elevated fat-free mass (e.g. muscle mass) is associated with increased lung function in the elderly [31]. Studies have thus shown an association between body fat and lung function. Nonetheless, to our knowledge, no previous study has investigated the importance of the separate BIA variables (trunk fat, arm, and leg fat) regarding its association with lung function. In our study, we wanted to determine which of the BIA measures drive this association. All BIA variables, that is, trunk fat, arms, and legs, were strongly correlated with each other and they all correlated with lung function. When BIA variables were analysed separately in relation to lung function, trunk fat is clearly the most important factor driving this association. To the best of our knowledge, no other study has demonstrated this through calculations. Thus, fat on the extremities can be disregarded when body fat is associated with lung function; rather, we should be focusing on trunk fat.

Furthermore, our results also confirm BMI is not interchangeable with BIA when assessing obesity. Nearly the same proportion of men and women in this study were obese according to their BMI. But our results showed a sex-based difference in obesity and body fat. Compared to men, women had a higher amount of fat mass, which was distributed differently in the body. The female subjects had a greater proportion of fat distributed on the hips and legs than men, while men had a greater proportion of abdominal fat. This is consistent with previous data which describe that men have more fat subcutaneously and viscerally compared to women [13, 14, 39]. This result is in accordance with previous studies showing that BMI is an inadequate measure when excess fat is associated with reduced lung function [4, 6, 10].

The reference value for spirometry was based on Caucasians [33]; however, 94% of the participants were of European descent, which leads to a small uncertainty in the spirometry data. However, as this represents only small proportion of a large study population, it is likely not to impact on the overall results is not significant. The advantages of this study include not only the large population but also that we have used data collected from one single study. This provides a continuity that is difficult to achieve consistency in data when collected from several different test centres. Furthermore, we have used the statistical analysis, WQS, which can distinguish between several strongly correlated variables. This has been vital when separating the impact of BIA variables and waist circumference on lung function.

There are both advantages and disadvantages to using waist circumference and BIA as methods to measure obesity in relation to lung function. As previously mentioned, waist circumference is a common measurement when assessing if an individual is overweight and a part of the definition of metabolic syndrome. However, it is also important that it be performed correctly. There are several studies that show uncertainty in the measurement, with poor reproducibility, especially in the case of obesity [21, 22, 40, 41]. It is necessary to obtain accurate and relevant information about patients to make an accurate assessment. Unfortunately, it has been shown that it is difficult to standardise waist measurements [40, 41]. Although there are established protocols for waist circumference measurement, it is not clear how well this is implemented in the clinic [21, 22]. BIA requires no training or experience and is easy to standardise. Furthermore, since it is digital, it can be linked directly to the patient’s record to avoid transmission errors. Since the method contributes with information about both abdominal and visceral fat, the method should be of clinical use. This is especially true regarding people with gynoid body shape, which is generally women. There are great advantages to using digital aids such as BIA in healthcare, and digital equipment is steadily becoming increasingly common, especially after COVID-19 [42].

The reliability of the BIA method has been disputed. However, the method has been further developed in recent years, with improvements in terms of technical conditions with multi-frequency and thus segment analysis, as well as refined prediction equations. This has meant increased measurement accuracy and reliability. Multi-frequency analysis distinguishes between several types of tissues, which contributes to a more accurate measurement [26, 28, 29]. Several studies have shown a good agreement between DXA and BIA, which makes multi-frequency BIA a useful alternative to DXA [16, 28, 43]. The data used in this study were based on one measurement per participant. The BIA method has previously shown some variability; therefore, repeated measurement values may have increased precision for this study.

## Conclusion

Our results show that even if waist circumference is important, trunk fat provides additional information and has a strong correlation to impaired lung function, especially for women. This could be of use in clinical setting and in further studies. We suggest that trunk fat should be considered when assessing the impact of adipose tissue on lung function and should potentially be included in health controls.

### Supplementary Information

Supplementary Material 1.

## Data Availability

All calculations were based on data extracted from the Epihealth database. Info on applications for data from EpiHealth can be made at https://www.epihealth.lu.se/en/cohort/researchers

## Abbreviations

BIA: Bioelectrical impedance analysis
BF%: Body fat percentage
BMI: Body mass index
COPD: Chronic obstructive pulmonary disease
DXA: Dual-energy X-ray absorptiometry
FM: Fat mass
FFM: Fat-free mass
FEV1: Forced expiratory volume in 1 s
FVC: Forced vital capacity
WQS: Weighted Quantile Sum Regression
