# Supplementary material for: Bioelectrical impedance and lung function—associations with gender and central obesity: results of the EpiHealth study
Source: BMC Pulm Med. 2024 Jul 4;24:319. doi: 10.1186/s12890-024-03128-0 (PMC11225376; doi:10.1186/s12890-024-03128-0)
Supplement: Supplementary file 1 — Supplementary Material 1. [file 12890_2024_3128_MOESM1_ESM.docx]

Supplement figure


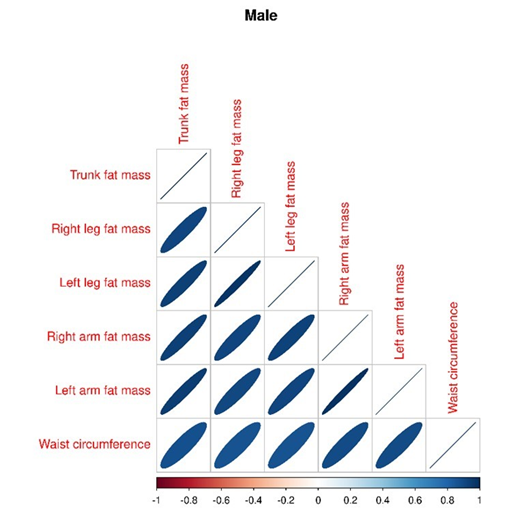


**Figure S1a.** Correlations between the BIA variables and waist measurement in men. The shape of the ellipse indicates the strength of the relationship, where a narrower ellipse corresponds to a stronger correlation.


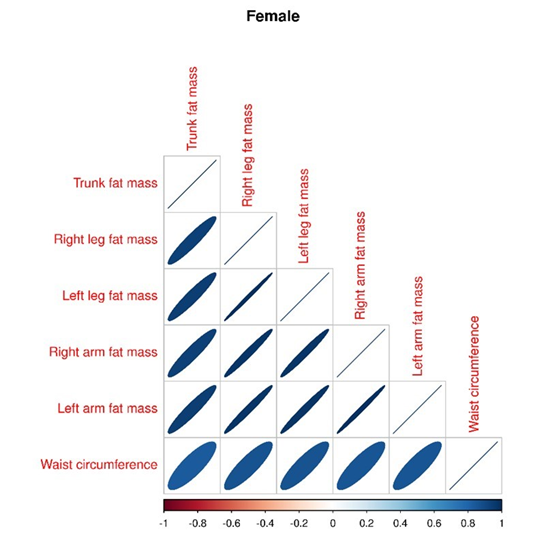


**Figure S1b.** Correlations between the BIA variables and WC in women. The shape of the ellipse indicates the strength of the relationship, where a narrower ellipse corresponds to a stronger correlation.

**
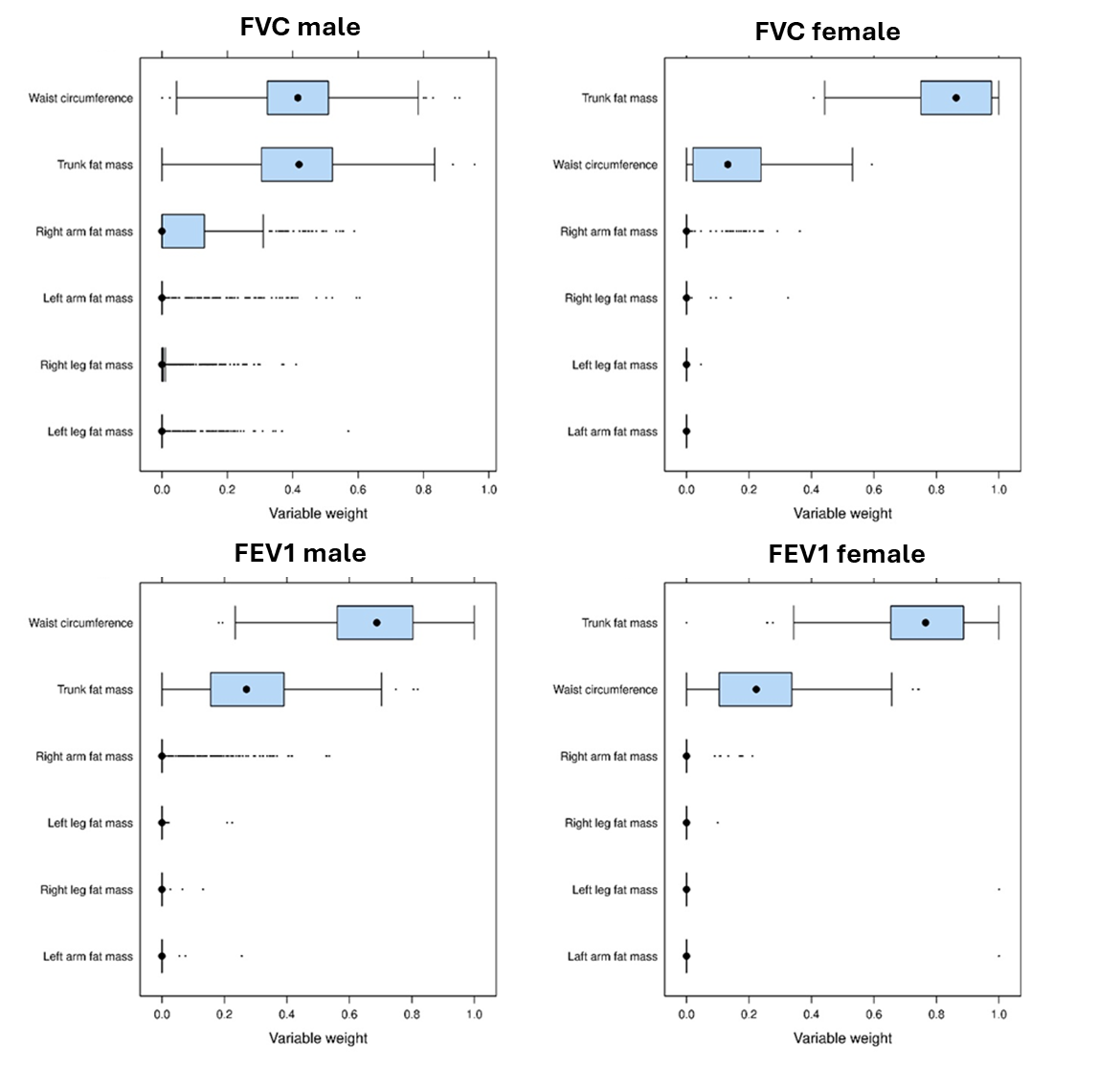
**

**Figure S2.** Boxplots of the bootstrap distribution of the variable weights from the BIA/waist index using weighted quantile sum regression adjusting for height, weight, age, education, physical activity, and smoking.
